# Fisetin, a phytochemical, potentiates sorafenib-induced apoptosis and abrogates tumor growth in athymic nude mice implanted with BRAF-mutated melanoma cells

**DOI:** 10.18632/oncotarget.5064

**Published:** 2015-07-31

**Authors:** Harish Chandra Pal, Ronald D. Baxter, Katherine M. Hunt, Jyoti Agarwal, Craig A. Elmets, Mohammad Athar, Farrukh Afaq

**Affiliations:** ^1^ Department of Dermatology, University of Alabama at Birmingham, Birmingham, AL, USA; ^2^ Comprehensive Cancer Center, University of Alabama at Birmingham, Birmingham, AL, USA

**Keywords:** melanoma, fisetin, sorafenib, cell proliferation, apoptosis

## Abstract

Melanoma is the most deadly form of cutaneous malignancy, and its incidence rates are rising worldwide. In melanoma, constitutive activation of the BRAF/MEK/ERK (MAPK) and PI3K/AKT/mTOR (PI3K) signaling pathways plays a pivotal role in cell proliferation, survival and tumorigenesis. A combination of compounds that lead to an optimal blockade of these critical signaling pathways may provide an effective strategy for prevention and treatment of melanoma. The phytochemical fisetin is known to possess anti-proliferative and pro-apoptotic activities. We found that fisetin treatment inhibited PI3K signaling pathway in melanoma cells. Therefore, we investigated the effect of fisetin and sorafenib (an RAF inhibitor) alone and in combination on cell proliferation, apoptosis and tumor growth. Combination treatment (fisetin + sorafenib) more effectively reduced the growth of BRAF-mutated human melanoma cells at lower doses when compared to individual agents. In addition, combination treatment resulted in enhanced (i) apoptosis, (ii) cleavage of caspase-3 and PARP, (iii) expression of Bax and Bak, (iv) inhibition of Bcl2 and Mcl-1, and (v) inhibition of expression of PI3K, phosphorylation of MEK1/2, ERK1/2, AKT and mTOR. In athymic nude mice subcutaneously implanted with melanoma cells (A375 and SK-MEL-28), we found that combination therapy resulted in greater reduction of tumor growth when compared to individual agents. Furthermore, combination therapy was more effective than monotherapy in: (i) inhibition of proliferation and angiogenesis, (ii) induction of apoptosis, and (iii) inhibition of the MAPK and PI3K pathways in xenograft tumors. These data suggest that simultaneous inhibition of both these signaling pathways using combination of fisetin and sorafenib may serve as a therapeutic option for the management of melanoma.

## INTRODUCTION

Incidence of melanoma, the most aggressive and lethal skin cancer, is rising rapidly worldwide [1]. An estimated 73,870 new cases and 9,940 deaths have been projected to occur in the United States in 2015. Approximately 75% of skin cancer-related deaths are caused by melanoma [2]. Moreover, very low survival rates of only 6 to 9 months have been reported in patients after visceral metastasis [3, 4]. Increased cell proliferation, enhanced cell survival and tumorigenesis are associated with mutational activation of a serine/threonine kinase BRAF in approximately 60% of melanomas [5, 6]. Substitution of valine with glutamic acid at position 600 (V600E) in BRAF results in tremendous increase in BRAF kinase activity leading to constitutive activation of the BRAF/MEK/ERK (MAPK) signaling pathway [5, 6].

Sorafenib is an orally active small molecule and multi-kinase modulator that inhibits the serine/threonine kinases CRAF, BRAF (wild type) as well as mutant BRAF^V600E^ [7, 8]. In addition, several studies have demonstrated that sorafenib inhibits activation of VEGR, PDGFR, FGFR, c-KIT, MET, MAPK and angiogenesis [8, 9]. Sorafenib also induces apoptosis *in vitro* and *in vivo* in tumor cells harboring BRAF and/or KRAS or NRAS mutations [8, 9]. Unfortunately, sorafenib demonstrated poor efficacy in melanoma patients when employed as a single agent [9, 10].

The PI3K/AKT/mTOR (PI3K) signaling pathway, in addition to MAPK, also plays a vital role in the growth, proliferation and survival of melanoma cells [11, 12]. The deletion or mutational inactivation of PTEN, which negatively regulates PI3K, has been reported in 10-30% of late-stage melanomas [13, 14]. Furthermore, the PI3K downstream effector protein AKT has exhibited overexpression in 50-75% of melanomas [15]. Recent studies have shown that the MAPK pathway also cooperates with PTEN-PI3K signaling to enhance cell proliferation, survival and tumor progression [13, 14]. This evidence suggests that it may be beneficial to target multiple signaling pathways in the treatment of melanoma. Therefore, the combination of RAF inhibitor sorafenib with pharmacologically active agents that target parallel signaling pathways may be a promising strategy to inhibit cell proliferation, survival and tumor progression.

Phytochemicals offer promising potential for the development of more effective strategies for the prevention/treatment of melanoma. Thus, identification of phytochemicals that can be used in combination with lower doses of chemotherapeutic drugs is of high clinical relevance. One such agent, fisetin, a naturally occurring flavonoid, is found in several fruits and vegetables, such as strawberries, apples, persimmons, grapes, onions and cucumbers. The anti-oxidative, anti-inflammatory and neuro-protective activities of fisetin have been reported in various studies [16-18]. It has exhibited anti-proliferative, pro-apoptotic and anti-tumorigenic activities against various cancers by inhibiting Wnt/β-catenin, PI3K/AKT/mTOR, and NFκB signaling pathways [19-23]. In our previous studies, we demonstrated that fisetin reduces melanoma cell invasion and epithelial to mesenchymal transition [22]. Murine investigations have also shown that fisetin was rapidly absorbed and detectable in serum [24-27]. To improve the efficacy of sorafenib in the treatment of melanoma, we studied combination therapy (fisetin and sorafenib) to evaluate whether fisetin potentiates sorafenib-mediated cell death and tumor growth inhibition. We found that combination treatment effectively inhibited BRAF-mutated melanoma cell growth, induced apoptosis, down-regulated MAPK and PI3K signaling pathways *in vitro* and *in vivo*. In addition, combination therapy greatly reduced angiogenesis markers in tumors of BRAF-mutated melanoma xenografts.

## RESULTS

### Fisetin potentiated sorafenib-mediated inhibition of cell growth, reduction of colony formation and induction of apoptosis in BRAF-mutated melanoma cells

**Figure 1:**
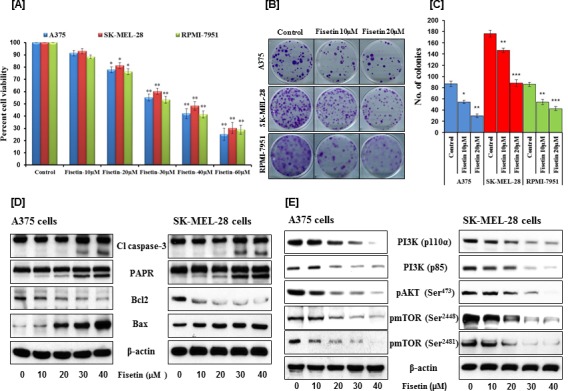
Effects of fisetin on cell viability, colony formation, apoptosis and on modulation of PI3K signaling pathway in BRAF-mutated melanoma cells BRAF-mutated melanoma cells (A375, SK-MEL-28 and RPMI-7951) were treated with the indicated concentrations of fisetin. **A.** The MTT assay was performed to determine the cell viability after 48 hrs of treatment. Data shown here are mean ± SEM of three separate experiments in which each treatment was repeated in 10 wells. *P < 0.05; **P < 0.01 versus control. **B.** & **C.** After treatment with fisetin for 24 hrs, the colony assay was performed by seeding melanoma cells in 6-well culture plates at a density of approximately 500 cells/well in 3 ml medium. Cells were allowed to grow in complete growth medium for 2 weeks before crystal violet staining. The data shown here are from a representative experiment repeated three times with similar results. *P < 0.05; **P < 0.01; ***P < 0.001 versus control. For Western blotting, after treatment with fisetin for 48 hrs, the effects on **D.** cleaved caspase-3, PARP and Bcl2 family proteins, **E.** modulation of PI3K signaling pathway was determined. Equal protein loading was confirmed by stripping the immunoblot and reprobing it for β-actin. The data shown here are from a representative experiment repeated three times with similar results.

Maintenance of uncontrolled proliferation and survival of malignant cells is the critical step for tumor initiation and progression [28]. Therefore, the effects of fisetin on short- and long-term growth of BRAF-mutated A375, SK-MEL-28 and RPMI-7951 melanoma cells were determined by an MTT and colony formation assays. Results of the MTT assay demonstrated that fisetin (10-60μM) treatment significantly decreased the growth of A375 (8.64-61.75%; *p* < 0.01), SK-MEL-28 (6.94-59.79%; *p* < 0.01) and RPMI-7951 (11.60-64.11%; *p* < 0.01) cells in a concentration-dependent manner (Figure 1A). At low concentrations, fisetin effectively inhibited long-term cell proliferation as shown by dose-dependent decrease in colony number and size (Figures 1B and 1C). At high concentrations, fisetin induced apoptosis in BRAF-mutated melanoma cells as evidenced by cleavage of caspase-3 and PARP, and modulation in Bcl2 family proteins (Figure 1D). Fisetin also inhibited protein expression of the PI3Kp110α and PI3Kp85 subunits and reduced phosphorylation of AKT at Ser^473^ (Figure 1E). We also observed that fisetin inhibited phosphorylation of mTOR at Ser^2448^ and Ser^2481^ residues (Figure 1E). These results illustrate fisetin’s abilities to inhibit melanoma cell growth and induce apoptosis by modulating the PI3K/AKT/mTOR (PI3K) signaling pathway.

**Figure 2:**
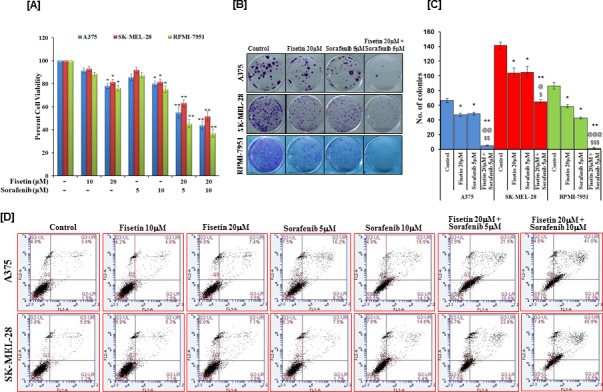
Effects of fisetin, sorafenib and their combinations on cell viability, colony formation and apoptosis of BRAF-mutated melanoma cells BRAF-mutated melanoma cells (A375, SK-MEL-28 and RPMI-7951) were treated with various concentrations of fisetin, sorafenib and their combinations. **A.** The MTT assay was performed to determine cell viability after 48 hrs of treatment. Data shown here are mean ± SEM of three separate experiments during which each treatment was repeated in 10 wells. *P < 0.05; **P < 0.01 versus control. **B**. & **C**. The colony assay was performed by seeding approximately 500 melanoma cells per well in 6-well culture plate after 24 hrs treatment with indicated concentration of fisetin, sorafenib and their combination. Cells were allowed to grow in complete growth medium for 2 weeks before crystal violet staining. The data shown here are from a representative experiment repeated three times with similar results. *P < 0.05; **P < 0.01 versus control. ^@^*p* < 0.05, ^@@^*p* < 0.01 and ^@@@^*p* < 0.001 versus fisetin treated group. ^$^*p* < 0.05, ^$$^*p* < 0.01 and ^$$$^*p* < 0.001 versus sorafenib treated group. **D.** The apoptosis assay was performed by treating the cells with fisetin, sorafenib and their combinations for 48 hrs as described in Materials and Methods section. The data shown here are from a representative experiment repeated three times with similar results.

To enhance therapeutic potential by simultaneously targeting two different pathways, fisetin was combined with the RAF inhibitor sorafenib. Twenty μM fisetin was combined with 5 and 10μM sorafenib to evaluate the effects of combination-therapy on short-term proliferation. MTT assay results demonstrated a significant reduction in cell growth inhibition (45-56% for A375; 36-48% for SK-MEL-28; and 55-63% for RPMI-7951 cells) in combination treatment groups to that when compared with their respective control. (Figure 2A). To further explore the observed inhibitory effects exhibited by combinations of fisetin and sorafenib, combination index (CI) values were determined. Values of CI (0.57, 0.814 and 0.507 for A375, SK-MEL-28 and RPMI-7951 respectively for combination of 20μM fisetin and 5μM sorafenib; and 0.44, 0.774 and 0.513 for A375, SK-MEL-28 and RPMI-7951 respectively for combination of 20μM fisetin and 10μM sorafenib) clearly demonstrated that combination treatment exerted synergistic growth inhibition of BRAF-mutated melanoma cells (Table 1). Since therapeutic efficacy of agents depends on long-term effects on cancer cells, we evaluated fisetin in combination with sorafenib on the colony formation abilities of melanoma cells. Results of the clonogenic assay clearly demonstrated that combination treatment effectively inhibited the colony number and size of BRAF-mutated melanoma cells (A375, SK-MEL-28 and RPMI-7951) (Figures 2B and 2C).

Furthermore, combination treatment (fisetin and sorafenib) was more effective at inducing apoptosis in A375 and SK-MEL-28 cells as compared to individual agents (Figure 2D). Treatment of A375 cells with 10 and 20μM of fisetin for 48 hrs resulted in 2.5% and 3.5% early apoptotic cell population and 4.8% and 7.4% late apoptotic cell population respectively. Similarly, treatment of A375 cells with 5 and 10μM of sorafenib for 48 hrs resulted in 6.2% and 14.1% early apoptotic cell population and 10.2% and 15.5% late apoptotic cell population respectively. Interestingly, the combination of 20μM fisetin with 5 or 10μM of sorafenib greatly enhanced the apoptotic cell population of A375 cells (25.5% and 16.3% early apoptotic population; 21.5% and 41% late apoptotic population) (Figure 2D). The observed increase in late apoptotic cell population following combination treatment demonstrated that combination treatment induced apoptosis more rapidly than monotherapy. In addition, fisetin (20μM) potentiated the apoptosis-inducing potential of sorafenib (5 or 10μM) in SK-MEL-28 cells since a higher apoptotic cell population (9.1% and 10.5% early apoptosis; 23.8% and 45.9% late apoptosis) was observed in these combination treatments than 10 and 20μM fisetin (2.1% and 2.8% early apoptosis; 5.3% and 7.1% late apoptosis) and 5 and 10μM sorafenib (1.7% and 5.4% early apoptosis; 7.5% and 14.6% late apoptosis) monotherapy-treated cells (Figure 2D). These results clearly demonstrated that fisetin potentiates sorafenib-mediated inhibition of cell proliferation and augments cell death by facilitating apoptosis.

**Table 1:** Combination index (CI) of fisetin and sorafenib on cell growth inhibition of BRAF-mutated melanoma cells

Treatments	Melanoma cell lines (% growth inhibition)			Combination Index (CI)		
	A375	SK-MEL-28	RPMI-7951	A375	SK-MEL-28	RPMI-7951
**Fisetin 10μM**	91.35	93.05	88.39			
**Fisetin 20μM**	77.99	81.33	76.25			
**Sorafenib 5μM**	85.68	92.02	87.58			
**Sorafenib 10μM**	80.02	81.59	75.25			
**Fisetin 20μM + Sorafenib 5μM**	54.86	63.19	45.25	0.57	0.814	0.507
**Fisetin 20μM + Sorafenib 10μM**	43.75	51.57	36.96	0.44	0.774	0.513

Combination index (CI) values for fisetin and sorafenib were calculated by Compusyn software (http://www.combosyn.com/feature.html). CI values were calculated from at least three separate experiments as described in Materials and Methods section. CI values <1 showed synergism, CI value >1 indicate antagonistic, CI value =1 suggests additive effects.

### Fisetin enhanced sorafenib-mediated cleavage of caspase-3 and PARP and modulated expression of Bcl2 family proteins in BRAF-mutated melanoma cells

**Figure 3:**
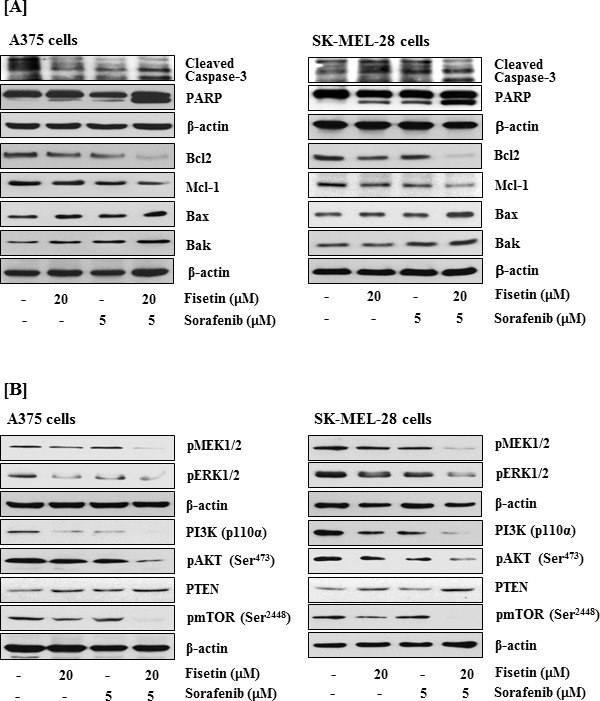
Effects of fisetin, sorafenib and their combination on cleavage of caspase-3 and PARP, expression of the Bcl2 family proteins, and on modulation of MAPK and PI3K signaling pathways in BRAF-mutated melanoma cells BRAF mutated melanoma cells (A375 and SK-MEL-28) were treated with fisetin 20μM, sorafenib 5μM and their combination (fisetin 20μM + sorafenib 5μM) for 48 hrs to determine their effects on **A.** cleavage of caspase-3 and PARP, and Bcl2 family proteins expression **B.** modulation in the MAPK and PI3K signaling pathways. Equal protein loading was confirmed by stripping the immunoblot and reprobing it for β-actin. The data shown here are from a representative experiment repeated three times with similar results.

Since higher concentrations of fisetin induced apoptosis in melanoma cells (Figure 1D), we next examined the effects of fisetin in combination with sorafenib at lower concentrations on activation of caspase-3, cleavage of PARP and expression of Bcl2 family proteins in melanoma cells. As shown in Figure 3A, the treatment of A375 and SK-MEL-28 cells with either 20μM fisetin or 5μM sorafenib demonstrated modest effects on activation of caspase-3 and cleavage of PARP. However, when these two agents were combined the cleavage of caspase-3 and PARP was greatly enhanced in both the cell lines. While monotherapy also exhibited modest effects on expression of anti-apoptotic proteins Bcl2 and Mcl-1, combination treatment more effectively reduced expression of these survival proteins. In addition, the combination was more effective at inducing expression of pro-apoptotic proteins Bax and Bak as compared to individual agents (Figure 3A).

### Fisetin in combination with sorafenib effectively down-regulated MAPK and PI3K signaling pathways in BRAF-mutated melanoma cells

Constitutive activation of MAPK signaling in melanoma is associated with increased cell proliferation, survival and tumor progression [29, 30]. Studies have demonstrated that activated BRAF in the MAPK signal transduction pathway propagates signals by phosphorylating MEK1/2 and ERK1/2 [31, 32]. Therefore, we investigated the effects of fisetin and sorafenib alone and in combination on MAPK signaling pathway. We found that combination treatment more effectively reduced phosphorylation of MEK1/2 and ERK1/2 in BRAF-mutated melanoma cells than individual agents (Figure 3B). In BRAF-mutated melanoma, the MAPK pathway cooperates with PI3K signaling to promote melanoma initiation and progression [30, 33]. Activation of PI3K signaling that regulates cell proliferation, survival and progression of tumor is a common genetic event in melanomas. The elevated PI3K signaling correlates with progression of tumors and has been observed in metastatic melanomas [11, 12]. PI3K/AKT-mediated activation of the downstream target mTOR results in cell proliferation and survival by regulating the translation of proteins involved in tumor progression [34, 35]. Therefore, the effect of fisetin in combination with sorafenib on the PI3K signaling pathway was evaluated in BRAF-mutated melanoma cells. Western blot analysis demonstrated that combination treatment significantly reduced the protein expression of PI3K (catalytic subunit p110α and phosphorylation of AKT at Ser^473^ (Figure 3B). Furthermore, loss of functional PTEN, a negative regulator of PI3K signaling, has been reported in more than half of all melanomas [33]. Since BRAF mutation and concurrent down-regulation (reduced expression or loss of function) of PTEN is common in melanomas [33, 36], we also quantified PTEN expression in BRAF-mutated melanoma cells following combination treatment. We observed that expression of PTEN was greatly enhanced in combination treated cells as compared to cells treated with individual agents (Figure 3B). In addition, combination therapy greatly reduced the phosphorylation of mTOR at Ser^2448^ as compared to individual agents (Figure 3B).

### Fisetin potentiated the sorafenib-mediated tumor growth inhibition of BRAF-mutated melanoma cells in athymic nude mice

**Figure 4:**
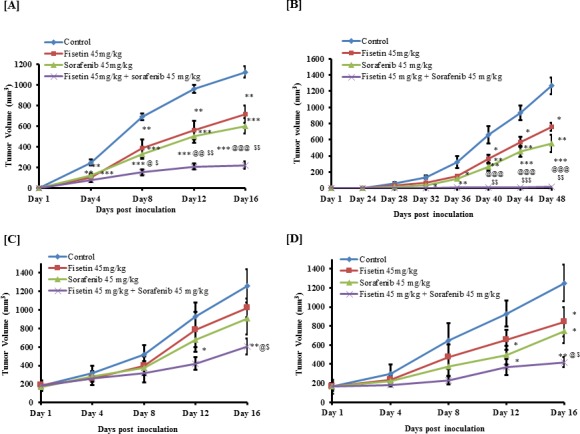
Effects of fisetin, sorafenib and their combination on tumor growth of subcutaneously implanted BRAF-mutated melanoma cells in athymic nude mice Athymic (*nu/nu*) female nude mice were subcutaneously injected with **A.** A375 cells **B.** SK-MEL-28 cells in each flank of mouse to initiate tumor growth. Mice were then randomly divided into four groups with 6 mice in each group. Twenty-four hours after cells implantation, mice were treated with fisetin, sorafenib and their combination as described in materials and methods section. Values represent mean ± SEM. *p < 0.05, **p < 0.01 and ***p < 0.001 versus control group. ^@^*p* < 0.05, ^@@^*p* < 0.01 and ^@@@^*p* < 0.001 versus fisetin treated group. ^$^*p* < 0.05, ^$$^*p* < 0.01 and ^$$$^*p* < 0.001 versus sorafenib treated group. Similarly, in another set of experiments **C.** & **D.**, mice were randomly divided into four groups with 6 mice in each group when their tumor size reached approximately 200 mm^3^. Mice were treated with test agents as described in materials and methods section. All mice were sacrificed when tumors reached a volume of ∼1200 mm^3^ in the control group. Average tumor volume of the control and treated groups was plotted over days after tumor cell inoculation. Values represent mean ± SEM. *p < 0.05, **p < 0.01 versus control group. ^@^*p* < 0.05 versus fisetin treated group. ^$^*p* < 0.05 versus sorafenib treated group.

We next determined the effects of fisetin, sorafenib and their combination on an *in vivo* xenograft mouse model subcutaneously implanted with BRAF-mutated A375 and SK-MEL-28 melanoma cells. The athymic nude mice implanted with melanoma cells were treated with fisetin (45 mg/kg body wt.), sorafenib (45 mg/kg body wt.) and a combination orally three times per week. The combination of fisetin and sorafenib showed significant higher tumor growth inhibition than the individual agents. Average tumor volume in mice injected with A375 cells and treated with vehicle was 1124.12 mm^3^; whereas in fisetin, sorafenib and combination treated groups, average tumor volume was 716.12, 602.50 and 224.03 mm^3^ respectively. This significant decrease indicated 36.29% (*p* < 0.01), 46.40% (*p* < 0.01) and 80.07% (*p* < 0.001) reduction in tumor growth respectively (Figure 4A). The growth inhibitory effects observed in combination group were significantly higher (*p* < 0.001 versus fisetin and *p* < 0.01 versus sorafenib) compared to monotherapy groups. In SK-MEL-28 cell-inoculated mice, the average tumor volume in the control group was 1265.69 mm^3^; whereas the fisetin, sorafenib and combination groups, averaged tumor volumes of 761.15, 552.02 and 15.93 mm^3^ respectively. This significant decrease indicated 39.86% (*p* < 0.01), 56.38% (*p* < 0.01) and 98.74% (*p* < 0.001) reduction in tumor growth respectively (Figure 4B). These results demonstrate that combination treatment inhibited tumor growth (*p* < 0.001 versus fisetin and *p* < 0.01 versus sorafenib) significantly higher than the fisetin and sorafenib montherapy groups.

Based on the results of our *in vitro* and *in vivo* growth inhibition studies, we hypothesized that a synergistic reduction in cell growth and induction of apoptosis with fisetin and sorafenib in BRAF-mutant melanoma cells might also abrogate growth of established tumors. Therefore treatment of mice was started when the tumor size reached ∼200 mm^3^ and terminated when tumor size reached ∼1200 mm^3^ in the control group. Monotherapy treatment (fisetin or sorafenib alone) of mice bearing A375 xenograft tumors resulted in substantial tumor growth reduction (18.88% and 28.16%) with 1022.54 and 905.53 mm^3^ respective tumor volumes when compared to control 1260.56 mm^3^. Combination treatment was highly effective in reducing tumor growth (56.65%; *p* < 0.01) as compared to the control group with tumor size of 546.36 mm^3^ only (Figure 4C). Furthermore, this effect was also significant (*p* < 0.05) when compared with fisetin or sorafenib alone. Similarly, the treatment of SK-MEL-28 tumor bearing mice with fisetin or sorafenib alone produced a significant tumor growth inhibition (32.56% and 40.55%; *p* < 0.05) with 844.59 and 744.41 mm^3^ tumor volume when compared with 1252.36 mm^3^ tumor of the control group. Combination of fisetin and sorafenib treatment produced highly significant tumor growth inhibition (66.43%; *p* < 0.01) with tumor volume of 420.37 mm^3^ only as compared to the control group. This effect was also significant (*p* < 0.05) when compared with tumor growth inhibitory effects of monotherapy (Figure 4D). These results clearly demonstrated that *in vitro* growth inhibitory effect of fisetin when combined with sorafenib was significantly translated under *in vivo* conditions.

### Fisetin augmented sorafenib-mediated inhibition of cell proliferation markers in xenograft tumors

**Figure 5:**
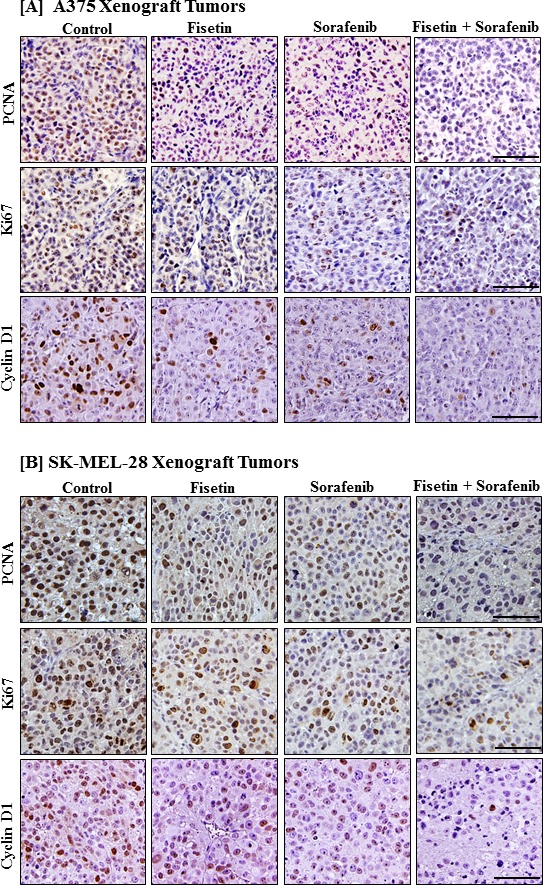
Effects of fisetin, sorafenib and their combination on markers of proliferation in tumor sections of athymic nude mice implanted with BRAF-mutated melanoma cells **A.** & **B.** Tumors from athymic nude mice implanted with melanoma cells and treated with vehicle or fisetin, sorafenib and their combination were immunostained for Ki67, PCNA and cyclin D1 expression. Photomicrographs show representative pictures from three independent tumor samples. Bar = 20μm.

Next, we examined the expression of molecules associated with cell proliferation in tumors of nude mice subcutaneously injected with BRAF mutated A375 and SK-MEL-28 melanoma cells following treatment with fisetin, sorafenib and the combination. Results from immunohistochemical analysis of tumor sections demonstrated a decreased intensity-staining pattern of cell proliferation markers such as PCNA, Ki67 and cyclin D1 in fisetin and sorafenib treated tumors as compared to their respective untreated control groups. Furthermore, this reduction was more pronounced in combination treatment (Figure 5A and 5B).

### Fisetin enhanced sorafenib-mediated apoptosis in xenograft tumors

**Figure 6:**
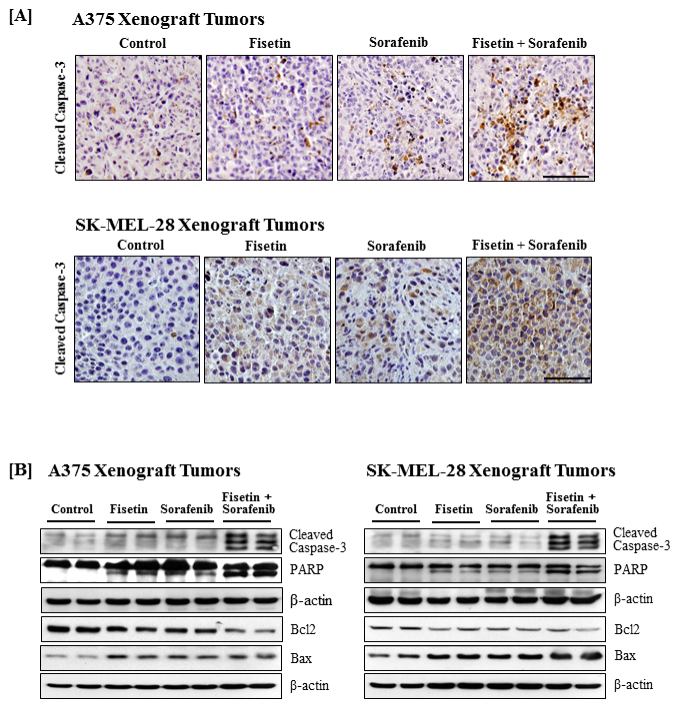
Effects of fisetin, sorafenib and their combination on cleavage of caspase-3 and PARP, and expression of Bcl2 family proteins in tumors of athymic nude mice implanted with BRAF-mutated melanoma cells Tumors from athymic nude mice implanted with melanoma cells and treated with vehicle or fisetin, sorafenib and their combination were harvested for immunostaining and Western blot analyses. **A.** Immunostatinng for cleaved caspase-3. Photomicrographs show representative pictures from three independent tumor samples. Bar = 20 μm. **B.** Western blotting for the cleavage of caspase-3 and PARP, and Bcl2 family proteins expression. Equal protein loading was confirmed by stripping the immunoblot and reprobing it for β-actin. The data shown here are from a representative experiment repeated three times with similar results.

Since fisetin enhanced the apoptosis-inducing potential of sorafenib *in vitro*, tumor sections and lysates were also evaluated for apoptosis. Immunohistochemical and Western blot analyses demonstrated that fisetin and sorafenib alone induced apoptosis in xenograft tumors. Combination therapy more effectively cleaved caspase-3 and PARP in xenograft tumors compared to individual agents (Figure 6A and 6B). In addition, Western blot analysis also revealed that combination treatment resulted in a greater decrease in the expression of anti-apoptotic protein Bcl2 with a concomitant increased expression of the pro-apoptotic protein Bax as compared to individual agents (Figure 6B).

### Fisetin potentiated sorafenib-mediated downregulation of the MAPK and PI3K signaling in xenograft tumors

**Figure 7:**
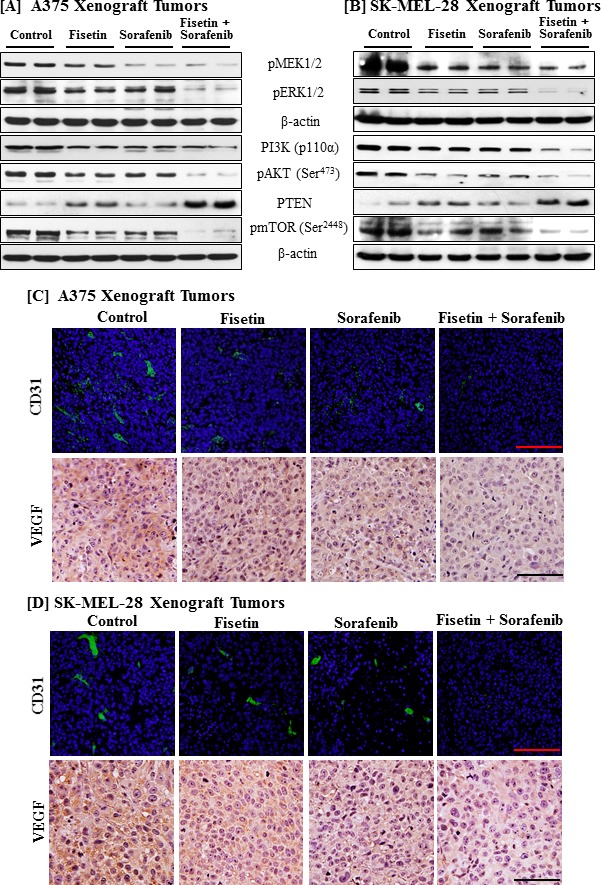
Effects of fisetin, sorafenib and their combination on MAPK and PI3K signaling pathways, and angiogenesis in tumor tissues of athymic nude mice implanted with BRAF mutated melanoma cells Tumors from athymic nude mice implanted with melanoma cells and treated with the vehicle or fisetin, sorafenib and their combination were harvested for immunostaining and Western blot analyses. **A.** & **B.** Protein lysates were examined for molecules of the MAPK and PI3K signaling pathways. Equal protein loading was confirmed by stripping the immunoblot and reprobing it for β-actin. The data shown here are from a representative experiment repeated three times with similar results. **C.** & **D.** Sections from tumors harvested form mice treated with vehicle or fisetin, sorafenib and their combination were stained for angiogenesis markers (CD31 and VEGF) as described earlier. Bar = 20μm.

Since results of *in vitro* and *in vivo* growth inhibition demonstrated that the combination of fisetin and sorafenib is highly effective in inducing apoptosis and reducing the tumor growth as compared to single agents, we examined the effects of these treatments on the MAPK and PI3K signaling pathways. Combination treatment was more effective in reducing the phosphorylation of MEK1/2 and ERK1/2 than either fisetin or sorafenib alone (Figure 7A and 7B). Immunoblot analysis of tumor lysates revealed that the combination of fisetin and sorafenib further reduced expression of PI3K, enhanced PTEN expression, and decreased phosphorylation of AKT and mTOR as compared to monotherapy (Figure 7A and 7B).

### Fisetin potentiated anti-angiogenic effects of sorafenib in xenograft tumors

Since tumors maintain their growth and survival by developing new blood vessels, agents targeting angiogenesis have shown promising anticancer potential. The combination of fisetin and sorafenib significantly inhibited melanoma cell growth, induced apoptosis and down-regulated cell survival signaling pathways *in vitro* and *in vivo*. An accumulating body of evidence has shown that agents targeting MAPK or PI3K signaling cascade also inhibit angiogenesis [37, 38]. Since combination treatment more effectively reduced MAPK and PI3K signaling pathways in xenograft tumors, we next determined the effects of combination therapy on angiogenesis in xenograft tumors. Figure 7C and 7D illustrate our findings that combination treatment was more effective in reducing the markers of angiogenesis (CD31 and VEGF) in tumors than monotherapy.

## DISCUSSION

In spite of exerting significant anti-cancer properties in various preclinical models, sorafenib, a multi-kinase inhibitor that targets MAPK signaling pathway, failed to translate its potential in melanoma patients due to limited efficacy against metastatic melanoma [9, 10]. Furthermore, patients treated with sorafenib exhibited considerable serious side-effects [39]. Although acquired resistance to BRAF inhibitors treatment is known to involve reactivation of MAPK signaling, an accumulating body of evidence suggests that activation of PI3K signaling also contributes to intrinsic cascades involved in melanoma development and progression [30, 33, 40, 41]. Therefore, it may be necessary to simultaneously target both pathways in the treatment of melanoma. In the present investigation, the non-toxic flavonoid fisetin has shown potential to inhibit melanoma cell growth and induce apoptosis of BRAF-mutated melanoma cells by reducing PI3K signaling. Therefore, fisetin was combined with sorafenib to enhance therapeutic efficacy. Our results demonstrated that the simultaneous targeting of these two parallel cell survival signaling pathways with fisetin and sorafenib resulted in enhanced cell growth inhibition, increased apoptosis and greater reduction in tumor growth and angiogenesis. Furthermore, the low-dose fisetin and sorafenib combination worked synergistically to inhibit melanoma cell growth and abrogate colony formation.

Evasion of apoptosis due to intrinsic or acquired resistance is one of the hallmarks of cancers resulting in tumor development and progression [42, 43]. The induction of apoptosis in cancer cells has been the center of traditional cancer chemotherapy and targeted therapy for many years [44, 45]. A growing body of evidence has demonstrated that sorafenib induces apoptosis in melanoma cells by activating pro-apoptotic proteins (Bad, Bak and Bax), down-regulating anti-apoptotic proteins (Bcl2, Bcl-Xl and Mcl-1) and inducing PARP cleavage [46, 47]. Our data demonstrated that fisetin also induces apoptosis in melanoma cells by cleavage of caspase-3 and PARP, and modulation of expression of the Bcl2 family of proteins in BRAF-mutated melanoma cells. When fisetin was combined with sorafenib, fisetin further augmented the apoptosis-inducing potential of sorafenib in BRAF-mutated melanoma cells. Several studies have clearly demonstrated that the MAPK and PI3K pathways intersect at multiple levels [30, 33, 40]. Constitutive activation of AKT protects cells from BRAF inhibitors, and detection of elevated AKT phosphorylation in BRAF-inhibitor-resistant melanoma cells suggests that enhanced AKT levels are associated with BRAF-inhibitor-ineffectiveness [40]. Activated AKT inhibits apoptosis by inactivation of pro-apoptotic proteins and cleavage of caspases. Furthermore, stability of anti-apoptotic protein Mcl-1 in melanoma cells is regulated by ERK1/2, and enhanced expression of this protein in melanoma cells has demonstrated resistance against BRAF inhibitor-induced apoptosis [46, 47]. Evidence suggests that ERK1/2 inhibition alone remains insufficient due to reprieve of ERK1/2-dependent negative feedback activation of RAS and PI3K signaling [48]. Therefore, successful inhibition of MAPK will require co-inhibition of PI3K and other alternative cell survival pathways. In the present investigation, we found that fisetin inhibited expression of PI3K and enhanced expression of PTEN as well as decreased phosphorylation of AKT and mTOR in BRAF-mutated melanoma cells. When fisetin was combined with sorafenib, co-targeting of these two parallel signaling pathways resulted in enhanced apoptosis in BRAF-mutated melanoma cells. In addition, fisetin alone reduced the phosphorylation of MEK1/2 and ERK1/2 in these cells. When combined with sorafenib, fisetin further potentiated the reduction in phosphorylation of MEK1/2 and ERK1/2. Furthermore, combination treatment also resulted in greater down-regulation of PI3K expression, and phosphorylation of AKT and mTOR as compared to fisetin or sorafenib alone. Fisetin also potentiated restoration of PTEN, a negative regulator of PI3K signaling that cooperates with MAPK signaling in melanoma.

More importantly, the *in vitro* effects of fisetin were translated under *in vivo* conditions. The addition of fisetin potentiated the antitumor activity of sorafenib against growing as well as pre-existing melanoma xenograft tumors. The BRAF mutation in melanoma drives enhanced proliferation through MAPK-induced expression of cyclin D1 and mediates BRAF inhibitor resistance in melanoma cells [49, 50]. In addition, PI3K signaling also regulates cyclin D1 expression and cell proliferation [34]. Like sorafenib, oral administration of fisetin monotherapy significantly inhibited the tumor growth of BRAF-mutated melanoma cells in nude mice by inhibiting tumor cell proliferation markers (PCNA, Ki67 and cyclin D1). Fisetin significantly inhibited tumor growth through induction of apoptosis by activating cleavage of caspase-3 and PARP proteins, and modulating expression of Bcl2 family proteins. In the present investigation, combination (fisetin and sorafenib) treatment produced a greater reduction in cell proliferation markers than either agent alone. Moreover, fisetin significantly potentiated sorafenib’s antitumor activity and apoptosis-inducing potential by further reducing MAPK and PI3K signaling.

Mounting evidence suggests that the formation of new blood vessels is a prominent feature among melanomas [51, 52]. Increased VEGF protein expression and accumulation in the tumor stroma has been associated with the transition of radial to vertical growth phase in melanoma [52, 53]. Several studies have demonstrated that mutant BRAF regulates secretion of pro-angiogenic factors, enhances growth and increases the development of vasculature in melanoma tumors [38, 54]. Studies have shown that sorafenib inhibits angiogenesis by inhibiting VEGF production and MAPK signaling in endothelial cells [9, 55]. In addition, PI3K/AKT/mTOR signaling also plays an important role in angiogenesis regulation in various cancers by increasing VEGF production in endothelial cells as well as in cancer cells [56, 57]. Therefore, fisetin likely augmented sorafenib’s anti-angiogenic properties in melanoma tumors by inhibiting PI3K pathway.

In summary, our findings demonstrate fisetin’s ability to potentiate the anti-proliferative, pro-apoptotic, and anti-tumor effects of sorafenib. This phytochemical may be a worthy, minimally-toxic adjuvant chemotherapy to prevent drug resistance and improve the therapeutic efficacy of anti-melanoma drugs in the future. Translational studies are the next steps to gauge the potential impact of these findings in humans.

## MATERIALS AND METHODS

### Reagents and antibodies

Fisetin (purity ≥ 98%), thiazolyl blue tetrazolium bromide (MTT) and mouse monoclonal β-actin antibody were purchased from Sigma-Aldrich (St. Louis, MO, USA). Sorafenib Tosylat N Mikron (BAY 43-9006) was provided by Bayer HealthCare (Bayer Pharma AG, Berlin, Germany). Dead cell apoptosis kit with annexin V alexa fluor^®^ 488 and propidium iodide (PI) were obtained from Life Technologies (Grand Island, NY, USA). Rabbit monoclonal or polyclonal antibodies for PARP, cleaved caspase-3, Bcl2, Bax, Bak, Mcl-1, PI3Kp110α, PI3Kp85, pAKT Ser^473^, pmTOR Ser^2448^, pmTOR Ser^2481^, pMEK1/2 and pERK1/2 were purchased from Cell Signaling Technology (Danvers, MA, USA). Rabbit monoclonal cyclin D1 antibody was purchased from Thermo Fisher Scientific Inc. (Rockford, IL, USA). Rabbit polyclonal Ki67, goat polyclonal PNCA, goat polyclonal PECAM-1/CD31 and mouse monoclonal VEGF antibodies were obtained from Santa Cruz Biotechnology, Inc. (Santa Cruz, CA, USA). Horseradish peroxidase conjugates goat anti-rabbit, rabbit anti-goat and rabbit anti-mouse antibodies were purchased from Millipore Corporation (Billerica, MA, USA). Alexa Fluor 488 rabbit anti-goat antibodies were procured from Life Technologies (Grand Island, NY, USA).

### Cell culture and treatment

Human malignant melanoma cells A375 (CLR-1619) and RPMI-7951 (HTB-66) were obtained from American Type Culture Collection (Manassas, VA, USA). SK-MEL-28 cells were obtained from Alan Houghton, Sloan-Kettering Institute for Cancer Research (New York, NY, USA). A375 and SK-MEL-28 cells were maintained in DMEM (Mediatech Inc., Manassas, VA, USA) and RPMI-1640 (HyClone Laboratories, Logan, Utah, USA) medium respectively supplemented with 10% heat-inactivated fetal bovine serum (Sigma-Aldrich Corporation, St. Louis, MO, USA) and 100 mg/ml penicillin-streptomycin (Mediatech Inc., Manassas, VA, USA). RPMI-7951 cells were cultured in EMEM medium (Quality Biologicals Inc., Gaithersburg, MD) supplemented with 10% heat-inactivated fetal bovine serum and 100 mg/ml penicillin-streptomycin. Cells were maintained at 37°C and 5% CO_2_ in a 95% humid environment. Fisetin and sorafenib used for the treatment of the cells were dissolved in DMSO (Fischer Scientific, Fair Lawn, NJ, USA). The final concentration of DMSO was ≤ 0.1% (v/v) in each treatment.

### Cell proliferation assay

To determine the growth inhibitory effect of fisetin on melanoma cells, approximately 1 x10^3^ melanoma cells per well were plated in 96-well plates (Becton Dickinson and Company, Franklin Lakes, NJ, USA) in complete growth medium. The following day, cells were treated with various concentrations of fisetin (10-60μM) for 48 hrs. After 48 hrs incubation cell viability was determined by employing MTT assay as described earlier [23]. In order to determine the effect of fisetin and sorafenib combination on cell growth, melanoma cells were treated with lower doses of fisetin (10 and 20μM), sorafenib (5 and 10μM), and their combinations (fisetin 20μM + sorafenib 5μM; and fisetin 20μM + sorafenib 10μM) for 48 hrs and the MTT assay was performed. Combination index (CI) values for fisetin and sorafenib were calculated by Compusyn software (http://www.combosyn.com/feature.html) developed by Chou-Talalay [58].

### Clonogenic assay

To determine the effect of fisetin on long term cell proliferation, 60-70% confluent melanoma cells were treated with indicated concentrations of fisetin for 24 hrs in complete growth medium. After incubation, cells from fisetin treated and control plates were harvested in separate tube. Approximately 500 cells per well from each tube were plated separately in 6-well plate (Corning Incorporated, Corning, NY, USA) in complete growth medium and allowed to grow for 2 weeks. Media was replaced after one week. After 2 weeks incubation, cells were washed once with chilled PBS (HyClone Laboratories, Logan, Utah, USA) and fixed in chilled methanol for 10 min. Cells were then stained with 0.5% crystal violet (Fischer Scientific, Fair Lawn, NJ, USA) solution prepared in 25% methanol for 5 min. To remove excess of crystal violet, wells were washed three times with water and air-dried. To determine the effect of combination treatment on colony formation, melanoma cells were treated with lower doses of fisetin/sorafenib/fisetin + sorafenib, and the colony formation assay was performed as described above.

### Apoptosis assay

Approximately 5×10^5^ cells were plated in 6-well plate. Next day, cells were treated with 10 and 20μM of fisetin, 5 and 10μM of sorafenib, and their combinations (fisetin 20μM + sorafenib 5μM; and fisetin 20μM + sorafenib 10 μM). After 48 hrs, apoptotic cell populations were determined by an Annexin V/Dead cell apoptosis detection kit obtained from Life Technologies (Grand Island, NY, USA) and analysed by Accuri C6 flow cytometer (Ann Arbor, MI, USA) as described earlier [23].

### Protein lysates preparation and western blotting

Melanoma cells were treated with 10-40μM fisetin alone for 48 hrs. Similarly, cells were treated with 20μM fisetin, 5μM sorafenib and a combination of 20μM fisetin and 5μM sorafenib for 48 hrs. After incubation, the medium was aspirated, and the cells were washed with PBS. Cells were lysed with ice-cold cell lysis buffer with freshly added protease inhibitor cocktail. Protein lysates from melanoma xenograft tumors were prepared with freshly frozen tumors samples. Approximately 250 mg tumor tissue was homogenized and lysed using 0.5ml of lysis buffer. Supernatant was collected from cell lysates after centrifugation at 14, 000 g for 10 min at 4°C [22]. Protein concentration of each sample was determined using the DC Protein assay kit (Bio-Rad Laboratories, Hercules, CA, USA). Western blotting was performed on 25-50μg protein using 8-10% Tris-glycine gels. Resolved protein was transferred onto a polyvinylidene fluoride membrane (Millipore Corporation, Billerica, MA, USA). The 5% non-fat dry milk in 0.1% Tween-20 in Tris-Buffered Saline (TBS), pH 7.6 was used for one hour to block non-specific sites on blots. Blots were incubated with the primary antibodies in blocking buffer overnight at 4°C followed by incubation with horse-radish peroxidase conjugate secondary antibodies. Bands on the membrane were developed by chemiluminescence using Pierce ECL Western Blotting Substrate reagents (Thermo Scientific, Rockford, IL) and autoradiography using HyBlot CL Autoradiography Film obtained from Densville Scientific Inc., (Metuchen, NJ, USA) [22, 23].

### Treatment of athymic nude mice

All *in vivo* tumor growth experiments were performed in accordance with the Institutional Animal Care and Use Committee (IACUC) guidelines and animal protocol (APN:140809459) used in this study was approved by the IACUC. Five-week-old female athymic nude mice were purchased from NCI-Frederick National Laboratory for Cancer Research and housed in a 12 hrs light/12 hrs dark schedule at 24±2°C temperature, 50%±10% relative humidity under pathogen-free conditions. Mice were fed with phytochemical free diet AIN-76 SEMI PD (Test Diet, Richmond, IN, USA) *ad libitum*. Mice were subcutaneously inoculated with 0.1ml of 2.5×10^6^ A375 cells or 5×10^6^ SK-MEL-28 cells (prepared in a 50μl media + 50μl matrigel) in each flank to initiate tumor growth. Mice were then randomly divided into two sets of four groups with 6 mice in each group. The first set of four groups was treated on the day following cell inoculation. In the first set, mice of group one were treated with 200μl vehicle orally and served as control. The mice of the second group received fisetin 45mg/kg orally in 200μl of 5% cremaphor EL + 2% ethanol in water, three times/week. The mice of the third group received sorafenib 45mg/kg orally in 200μl of 5% cremaphor EL + 2% ethanol in water, three times/week. The mice of the fourth group received a combination of fisetin 45mg/kg + sorafenib 45mg/kg orally in 200μl of 5% cremaphor EL + 2% ethanol in water, three times/week. Tumor sizes were measured twice a week, and tumor volume was calculated by the formula ½ (*L*1 × *L*2 × *H*), where *L*1 is the long diameter, *L*2 is the short diameter, and H is the height of the tumor. In second set of experiment, mice were treated similarly as described above when their tumor size reached approximately 200 mm^3^. The experiment was terminated by sacrificing all the mice by CO_2_ inhalation and death was confirmed by cervical dislocation when tumor volume reached approximately 1200 mm^3^ in the control group.

### Immunohistochemical and immunofluorescence staining

Tumors from the mice were harvested, fixed in 10% neutralized formalin and embedded in paraffin. For immunohistochemical and immunofluorescence staining, five micrometer thin sections were gradually deparaffinized in xylenes and rehydrated in ethanol. Antigen retrieval was performed by heating the tissue at 95°C for 30 min in citrate buffer (pH 6.0). After treatment with blocking buffer for 30 min at room temperature, sections were incubated with primary antibodies, subsequently incubated with secondary antibodies and analyzed for staining as described previously [22].

### Statistical analysis

The results are expressed as the mean ± SEM. Statistical analysis of all the data was performed by Student’s *t*-test. The *p* value < 0.05 was considered statistically significant.
